# Some patients (and some of us) respond better to some biological therapies: the as yet unsolved conundrum

**DOI:** 10.1186/s10195-018-0505-z

**Published:** 2018-08-20

**Authors:** Isabel Andia, Nicola Maffulli

**Affiliations:** 1 Regenerative Medicine Laboratory, BioCruces Health Research Institute, Cruces University Hospital, 48903 Barakaldo, Spain; 2 Department of Musculoskeletal Disorders, University of Salerno School of Medicine and Dentistry, Salerno, Italy; 3 Queen Mary University of London, Barts and the London School of Medicine and Dentistry, London, UK

## Abstract

Very often, treatment for many common musculoskeletal conditions is only palliative, or involves surgery with major shortcomings. Biological interventions—in particular, platelet-rich plasma (PRP) therapies—may well provide more effective treatments, but their actual efficacy is under scrutiny. PRP is biologically unique to each individual depending on endogenous and exogenous factors, including, but not limited to, demographic factors (i.e. age), immune status (i.e. microbiota), metabolic diseases and concomitant medications. All these potential modifiers of the ultimate effects of PRP have been poorly explored, and their relationship with efficacy has not been established.

## Introduction

Tendinopathies and osteoarthritis (OA) are debilitating conditions. Available treatments merely address symptoms and are therefore only palliative or involve surgery with major shortcomings. The opportunity of biological interventions—in particular, platelet-rich plasma (PRP) therapies—to provide more effective treatments is under scrutiny. In contrast with the traditional therapeutic approach based on one specific target, the PRP approach is based on complete modification of the biological milieu [1, 2]. Natural wound healing is initiated by platelets in fibrin clots, confirming their suitability for tissue repair and ability to provide cells with a suitable regenerative environment. PRP is variedly used across specialties and pathologies [3, 4]. However, despite a lack of safety concerns, routine use of PRP is not recommended in daily practice by public bodies because of unpredictability of its therapeutic effects. PRP can decrease pain and improve function in selected patients, as assessed by standardized dedicated questionnaires [5]. At present, patients are treated following the traditional “trial-and-error” approach to disease: we cannot predict which patients will have a positive response to PRP.

Conventional outcome scores only provide a vague idea of the potential anti-inflammatory and regenerative effects on either nociceptive and/or neurogenic inflammation. It is still challenging to assess in a practical and realistic time frame whether changes are occurring within the tissue/organ at a cellular or molecular level after PRP treatment. Development of tools combining demographic, genetic and metabolic data, and stage-related clinical characteristics could help in identifying candidate patients, thus improving the efficacy of these biological interventions.

## Is my patient a good candidate for PRP therapy?

The quality of PRP is based on an individual biological uniqueness, which varies depending on endogenous and exogenous factors, including, but not limited to, demographic factors (i.e., age), immune status (i.e., microbiota), and metabolic diseases including diabetes, obesity, or hyperuricemia, as well as concomitant medications. All these potential modifiers of the ultimate effects of PRP have been poorly explored, and their relationship with efficacy has not been established.

Describing responders versus nonresponders to PRP therapy to personalize their treatment strategy relies on scientific breakthroughs in our understanding of how a patient profile makes them a responder to PRP intervention. Given the molecular complexity of PRPs, it is unlikely that a single cytokine may provide sufficient patient discrimination. In fact, PRP actions are more complex than just the release of GFs. Recent proteomic data have identified a total of 1507 unique proteins in resting platelets, including 190 membrane-associated and 262 phosphorylated proteins [1]. Also, PRP contains hundreds of active component molecules with conflicting functions, an essential feature to maintain tissue homeostasis in different environments. The mixture has evolved through Darwinian selection and can be considered as having buffer properties, since its effects can be modulated by the recipient’s tissues. In this scenario, no single cytokine can predict the probability of response or be indicative of its quality. More broad approaches would include first assessing factors involved in PRP quality, and second the individual characteristics of the recipient tissue (i.e., inflammation, angiogenic state, cellularity etc.).

At least four features can help describe PRP quality. First are demographic factors, i.e. old vs young: Age-associated interactions and their clinical relevance are difficult to disentangle, but exposure of an aged organism to a youthful systemic environment can help identify whether circulating factors from the young organism can alter tissue function of the old or vice versa [6]. The model of heterochronic parabiosis, consisting of the surgical union of two organisms of different ages which then share blood circulation, allows the study of how plasma from a young animal can ameliorate a given pathology in the older [7]. In these experiments, tissues from heterochronic pairs are compared with those of isochronic pairs (two organisms of the same age sharing blood circulation) as a control: young blood plasma was able to ameliorate Alzheimer pathology [8]. Plasma appears to target molecular pathways involved in learning, memory, and inflammation. These findings suggest that age-associated impairment in cell function is induced to a significant extent by the molecular composition of the surrounding niche rather than by intrinsic cell changes alone.

Second, associated metabolic diseases should be considered as potentially PRP-modifying factors. Current research suggests that metabolic diseases impact on the vulnerability to develop tendinopathy or OA [9, 10]. Collagen mechanical and functional properties can be adversely altered by posttranslational protein adducts and crosslinks formed by advanced glycation end-products [11]. The latter may play a crucial role in the development of diabetes complications, such as tendinopathy or OA. Yet, most research indicates that PRP is successful in treating diabetic foot ulcers [12]. Similarly, we were not able to identify any difference regarding paracrine angiogenic or inflammatory actions between hyperuricemic PRP and normal PRP in tenocytes [13]. Moreover, we are still investigating whether systemic factors, such as circulating leptin involved in the subclinical inflammation present in obesity, influence the clinical efficacy of PRPs.

Third, the unique immune status of each patient may be relevant when selecting treatment. Microbiota are crucial for immunologic, hormonal, and metabolic homeostasis of their host. The synonymous term microbiome describes either the collective genomes of the microorganisms that reside in an environmental niche or the microorganisms themselves. There are >39 trillion bacteria in the human organism, and the bacterial DNA profile constitutes the microbiome, which is a unique fingerprint. Changes in microbiota are associated with wound healing [14]. Moreover, the effects of PRP in tendon healing is related to microbiota in an experimental Achilles-tendon-healing model [15].

Fourth, the presence of concomitant systemic medications in circulating plasma, including statins, quinolone antibiotics, corticosteroids, or aromatase inhibitors can be detrimental because of neurotoxicity. Moreover, monoamide local anesthetics, such as lidocaine, are often administered to enhance patient and physician comfort during the procedure. In fact, in a recent meta-analysis assessing the efficacy of PRP in tendon interventions, all protocols involved prior administration of 1–2 ml of local anesthetic [16]. Although administered perilesionally, the cytotoxicity of these products can compromise cell viability [17].

Finally, the clinical effect after PRP administration is a result of interaction with the host tissue. Thus, the status of the recipient tissue (i.e., reactive versus degenerative), including structural and biological abnormalities of the adjacent structures, should be described.

## The need of patient stratification

Novel findings in genetic biomarkers can be informative regarding the pathogenesis of osteoarthritis or tendinopathy and help to develop effective treatments. Given this background, the response to PRP treatment in relation to genetic variation can be explored using a gene-candidate approach. For example, rotator cuff tears are associated with enzymes involved in collagen degradation, including matrix metalloproteinase (MMP)1 and 3; in particular, polymorphisms associated with rotator cuff disease were identified in their gene promoter regions [18]. Other candidate genes also involved in the integrity of extracellular matrix, including tenascin (TNC) and Col5A1, show an association with tendinopathy in specific cohorts [19, 20]. These gene polymorphisms could be used as surrogate markers in guiding patient stratification and response to PRP treatment in different anatomical locations.

In OA research, findings using the candidate gene approach showed mutations of mitochondrial DNA in some cohorts, and these biomarkers were proposed as contributors to the risk of progression of knee OA [21]. Mitochondrial function is altered in osteoarthritic chondrocytes and may be related to chondrocyte apoptosis. Moreover, the identification of relevant endophenotypes (such as reduced intra-articular space or other structural biomarkers) can help to further characterize patient subgroups [22] and eventually help identify responder patients for PRP treatments by producing personalized treatment algorithms.

Limitations in PRP therapies give us the opportunity to explore novel ways to improve them, considering that endogenous (demography, comorbidities) and exogenous (medications) factors are potential modifiers of the (beneficial) actions of PRP. If we are to follow the concepts of personalized medicine, we need to define different causes of a condition, its course, and therapeutic response indicators that can predict the probability or quality of the response to PRP interventions.
